# Abnormal Presentation of Severe Hyponatremia

**DOI:** 10.7759/cureus.50883

**Published:** 2023-12-21

**Authors:** Alison Cullin, Andrew Trom

**Affiliations:** 1 Department of Emergency Medicine, Inspira Medical Center Mullica Hill, Mullica Hill, USA

**Keywords:** hyponatremia, generalized weakness, osmotic demyelination syndrome (ods), emergency condition, beer potomania syndrome, profound hyponatremia

## Abstract

Hyponatremia can be a life-threatening condition. Our patient presented alert and oriented, with bilateral upper and lower extremity weakness and gastrointestinal concerns. Labs demonstrated significant hyponatremia at 103 mEq/L, which is inconsistent with her lack of neurological symptoms. It is very rare to have such severe hyponatremia without altered mentation, seizures, or coma. Careful correction of sodium must be completed, and if it is done too quickly, there is a risk of osmotic demyelination syndrome. This makes hyponatremia and its treatment of the utmost importance.

## Introduction

Hyponatremia is defined as a serum sodium level less than 135 mEq/L (mild variations based on laboratory scales, reference range: 135-145 mEq/L) [1]. Most commonly, hyponatremia is associated with the inability to appropriately excrete water. While many organs are affected by changes in serum sodium concentration, the brain is especially sensitive to such changes. Those at risk of severe complications of hyponatremia include patients with renal failure, congestive heart failure, endocrine disorders, patients with alcohol use disorders, and those taking certain medications such as thiazide diuretics and angiotensin-converting enzyme (ACE) inhibitors or angiotensin receptor blockers (ARBs) [1]. A notable subcategory of hyponatremia is beer potomania. Initially reported in 1972, it is described as the excessive intake of alcohol (especially beer) combined with poor dietary solute intake. Beer is a particular culprit since it also has a low solute content (a sodium concentration of 1.8 mEq/L) [2]. If left untreated, severe hyponatremia can lead to significant complications, including seizures, obtundation, coma, and respiratory arrest. Here is an unusual presentation of a case of severe hyponatremia occurring in a patient with chronic beer consumption.

## Case presentation

A 59-year-old female with a medical history of coronary artery disease, chronic pain syndrome, hyperlipidemia, hypertension, neuralgia, tobacco use, and alcohol dependence presented to the emergency department (ED) with nausea, vomiting, diarrhea with bright red blood per rectum, and bilateral arm and leg weakness for the past four days. She endorsed abdominal cramping and reported having had a couple of falls over the past one to two days due to her leg weakness. The patient also endorsed neuralgias for bilateral feet. She denied fevers, melena, hematemesis, seizures, syncope, chest pain, and shortness of breath. The patient reported drinking six to 10 beers per day, smoking cigarettes, and using marijuana daily.

Initial vital signs on presentation were a temperature of 36.2 °C, a heart rate of 63 beats per minute (bpm), a respiratory rate of 17 breaths per minute, a blood pressure of 82/48 mmHg, and a pulse-oximetry of 96% on room air. No code stroke was called as symptoms had been present for four days. She was alert and oriented x4, in no acute distress, and appeared non-toxic. The patient was noted to have mildly dry mucous membranes. She had mild tenderness to palpation of the mid and lower abdomen and bilateral lower extremities. In addition, there were areas of ecchymosis in the bilateral lower extremities, right buttock, and back. No focal neurological deficits were found on examination.

The patient was given a 500-cc normal saline (NS) intravenous (IV) bolus, which improved her blood pressure to 100/61 mmHg. Lab evaluation demonstrated mild anemia with hemoglobin 11.5 g/dL, without leukocytosis. The chemistry panel demonstrated severe hyponatremia at 103 mEq/L and hypoglycemia at 65 mg/dL. The patient was given crackers and orange juice to improve glucose, as her mentation was appropriate. Other notable lab results are displayed in Tables 1-2.

**Table 1:** Initial laboratory results from the emergency department

Lab	Result	Reference range	Lab	Results	Reference range
Hemoglobin	11.5 g/dL	11.0 – 15.2 g/dL	Phosphorus	6.1 mg/dL	2.4 – 5.1 mg/dL
Creatinine	2.9 mg/dL	0.55-1.02 mg/dL	Alkaline phosphatase	772 U/L	46-116 U/L
Blood urea nitrogen (BUN)	48 mg/dL	9-23 mg/dL	Aspartate aminotransferase (AST)	307 U/L	0-34 U/L
Potassium	4.7 mEq/L	3.5-5.1 mEq/L	Alanine transaminase (ALT)	159 U/L	10-49 U/L
Sodium	103 mEq/L	136-145 mEq/L	Total bilirubin	2.2 mg/dL	0.3-1.2 mg/dL
Chloride	77 mEq/L	98 -107 mEq/L	Serum osmolality	221 mOsm/kg	275-295 mOsm/kg
Glucose	65 mg/dL	74 – 106 gm/dL	Magnesium	1.4 mg/dL	1.6-2.6 mg/dL
Carbon dioxide (CO_2_)	15 mEq/L	22-30 mEq/L	Thyroid-stimulating hormone (TSH)	5.393 mclU/mL	0.55 -4.78 mcIU/mL
Calcium	6.6 mg/dL	8.4-10.2 mg/dL	Hemoccult	Positive	Negative

**Table 2:** Urine electrolyte reports from the emergency department

Urine electrolytes	Result	Reference range
Sodium	<10 mEq/L	30-90 mEq/L
Potassium	12 mEq/L	0-40 mEq/L
Chloride	22 mEq/L	20-40 mEq/L
Creatinine	82 mg/dL	20-275 mg/dL
Urine osmolality	217 mOsm/kg	50-1,200 mOsm/kg
Specific gravity	1.010	1.001-1.030

A CT of the abdomen and pelvis without contrast demonstrated hepatic steatosis, which was otherwise unremarkable and noncontributory to her presentation.

The findings were consistent with hypotonic hyponatremia, likely associated with the patient’s chronic beer consumption. The patient was immediately placed on strict IV and by-mouth (PO) fluid restrictions. While the patient was in the ED, there were no changes in neurological status on multiple reevaluations. The patient was admitted to the intensive care unit (ICU).

By hospital day two, the patient had developed increasing confusion and somnolence. Based on extensive hyponatremia and mentation changes, nephrology was consulted. Per nephrology, it was recommended for the patient to have fluid restrictions and continue slow repletion via half-normal saline and desmopressin if needed to progress to the appropriate serum sodium goals they provide each day. Abdominal and retroperitoneal ultrasounds were unremarkable. The patient underwent a basic metabolic panel every four hours to monitor sodium. Of note, the patient did have increased sodium levels from 112 to 130 mEq/L within 24 hours. She then required a total of five doses of 2 mcg of desmopressin over three days to slow the elevation of sodium. By hospital day seven, the patient’s sodium had normalized to 138 mEq/L. The patient did have a gradual improvement in mentation as the sodium level improved. The patient had no respiratory depression or seizure activity associated with her severe hyponatremia. The patient was discharged home at a time of improved sodium and mentation.

## Discussion

Severe hyponatremia can have significant morbidity. It is also the most common electrolyte abnormality. It is especially common with patients with chronic alcohol use disorder if they consume low-solute beverages such as beer. The initial evaluation of hyponatremia consists of determining osmolality and overall volume status [3]. A majority of clinical hyponatremia cases are associated with low serum osmolality. In such patients, symptoms generally include generalized weakness, muscle cramps, nausea/vomiting, fatigue, headaches, mental status changes, and eventual seizures, coma, and respiratory arrest [2]. For hyponatremia associated with beer potomania, treatment includes nothing by mouth for 24 hours, avoiding IV hydration unless the patient is symptomatic, and using a finite amount of hydration, such as the recommended <800 mL/day [2, 4]. In chronically hyponatremic patients, furosemide 20 mg IV daily can also be given to assist with decreasing free fluid, thus increasing sodium concentration [4]. Initially, sodium levels will need to be repeated every two hours for close monitoring to prevent rapid overcorrection. Desmopressin or dextrose can be used to minimize free water excretion and slow the increase of sodium. The desmopressin dose is normally 2-4 mcg/dose IV. The patient will need to be admitted to the ICU if they have symptoms of severe hyponatremia or if their serum sodium concentration is less than or equal to 120 mEq/L.

Careful correction of sodium levels will be required. A conservative goal would be to increase serum sodium concentrations to less than 8-10 mEq/L in 24 hours. If corrected too quickly, patients can develop osmotic demyelination, which can cause irreversible neurological damage, including locked-in syndrome. This phenomenon is caused by the osmotic shift based on the solute concentration difference between the vasculature and brain parenchyma. The brain is unable to accommodate acute changes in tonicity, which leads to dreaded complications, including locked-in syndrome, when hyponatremia is corrected too rapidly [3]. These concerns are exacerbated in the cases of patients with chronic hyponatremia, such as our patient. During treatment of chronic hyponatremia, it is recommended to increase sodium concentrations on the lower end of the range, such as 8 mEq/L, sometimes even going down to 6 mEq/L, in 24 hours. If the patient is critically unstable with seizures and respiratory depression or arrest, it is appropriate to give a 100-mL bolus of 3% hypertonic saline every 10 minutes until seizure activity stops; however, this does increase the risk of developing osmotic demyelination syndrome [3,4].

Our patient’s presentation was unique in that she remained alert and oriented x4 with other symptoms consistent with mild hyponatremia, while her measured serum sodium was severely depleted at 103. It is abnormal for patients with such diminished concentrations of sodium to be without focal neurological deficits, altered mentation, and/or respiratory depression. Such a presentation was consistent with the notable chronicity of her hyponatremia, which had very slowly declined, allowing her cells to gradually adjust to the osmotic changes. This prevented her from developing the common symptoms associated with severe hyponatremia.

## Conclusions

In conclusion, hyponatremia is one of the most common electrolyte derangements and one of the most life-threatening conditions, especially when it becomes severe with a serum sodium level less than 120 mEq/L. Our case demonstrated that hyponatremia can present differently between chronic and acute hyponatremia. The patient presented with severe hyponatremia with a serum sodium level of 103 mEq/L without seizures or encephalopathic changes. She required a very slow correction because of her known alcohol use, suggesting the chronicity of her severe hyponatremia. When her serum sodium levels were rising too quickly, she required multiple doses of desmopressin to prevent the possible development of osmotic demyelination. Thus, severe hyponatremia is an electrolyte derangement that can vastly differ in presentation and morbidity if not appropriately considered.
